# Gut Microbiota Regulates the Homeostasis of Dendritic Epidermal T Cells

**DOI:** 10.3390/life14121695

**Published:** 2024-12-21

**Authors:** Jinwoo Chung, Joo-Chan Lee, Hanna Oh, Yesung Kim, Suin Lim, Chanu Lee, Yoon-Gyu Shim, Eun-Chong Bang, Jea-Hyun Baek

**Affiliations:** Laboratory of Inflammation Research, School of Life Science, Handong Global University, Pohang 37554, Republic of Korea; 21900683@handong.ac.kr (J.C.); joochan@kaist.ac.kr (J.-C.L.); hannaoh@handong.ac.kr (H.O.); 21800131@handong.ac.kr (Y.K.); lsi12312@handong.ac.kr (S.L.); 22000587@handong.ac.kr (C.L.); simbadanny@handong.ac.kr (Y.-G.S.); 22432003@handong.ac.kr (E.-C.B.)

**Keywords:** skin immunity, gamma-delta T cells, skin inflammation, dendritic epidermal T cells

## Abstract

Dendritic epidermal T cells (DETCs) are a γδ T cell subset residing in the skin epidermis. Although they have been known for decades, the fate of DETCs has largely remained enigmatic. Recent studies have highlighted the relationship between the gut microbiome and γδ T cells in various epithelial and non-epithelial tissues, such as the small intestine, lung, liver, gingiva, and testis. While the skin microbiota has been shown to impact skin γδ T cells, a direct relationship between the gut microbiota and DETCs remains unexplored. In this study, we investigated whether DETCs are regulated by the gut microbiota in the steady-state skin epidermis. We examined the occurrence of DETCs in Balb/c mice, which have a skin epidermis barely populated with DETCs, compared to C57BL/6 mice, under different housing conditions. Our findings reveal that local skin inflammation markedly increases DETC numbers in the ear epidermis of Balb/c mice and that DETCs are activated by environmental factors. Furthermore, an investigation of the gut microbiota under different housing conditions revealed distinct microbial compositions and functional profiles. Taken together, these results suggest a strong connection between DETCs and gut microbiota.

## 1. Introduction

Dendritic epidermal T cells (DETCs) in mice and epidermal resident γδ T cells in humans are distinct subsets of T cells that reside in the skin epidermis [1]. While most T cells (>90%) acquire T cell receptors (TCRs) with variable α (Vα) and β (Vβ) chains during development in the thymus, only a small fraction (<10%) differentiate into γδ T cells expressing variable γ (Vγ) and δ (Vδ) segments. Among these γδ T cells, DETCs are a unique subset known for their presence in the skin epidermis and expression of monomorphic Vγ5Vδ1 [2,3]. However, the specific ligands of these invariant TCRs are not fully understood.

In mice, DETCs constitute the majority of T cells in the skin epidermis (~90%), whereas the human skin epidermis contains a diverse range of T cells, with αβ T cells being the predominant type [4,5]. This has led to the belief that human skin lacks a counterpart to murine DETCs. However, recent evidence suggests that the diverse T cell landscape in human skin reflects the continuous exposure to environmental and pathogenic factors, such as ultra-violet (UV) light, which leads to the accumulation of regulatory and memory T cells [6,7]. Studies have also shown that the repeated irritation of mouse skin can alter the epidermal T cell composition, increasing the presence of memory T cells [8]. Additionally, αβ T cells have been found to functionally replace DETCs in murine skin when DETCs are depleted [9].

Despite their minor presence in humans, DETCs are known to play versatile roles in skin immunity, though these roles can also be ambiguous, and even contradictory. DETCs have been linked to both pro- and anti-inflammatory functions. They are suggested to contribute to inflammation by producing inflammatory cytokines (e.g., GM-CSF, IFN-γ, IL-2, IL-17, TNF-α) and attracting other immune cells to damaged sites (e.g., CCL-3, -4, -5, XCL1) [10,11,12,13,14]. DETCs are also implicated in wound repair [4,15,16,17] and anti-tumor immunity in the skin [18,19]. DETCs have been shown to help maintain skin homeostasis by promoting the maturation of Langerhans cells [20], supporting keratinocyte proliferation [21], and limiting DNA damage in keratinocytes [22]. Although DETCs are typically stable in their location, they can migrate within the epidermis during inflammation [23]. Unlike dermal γδ T cells, DETCs do not appear to migrate to skin-draining lymph nodes or recirculate [24,25].

DETCs are believed to enter the epidermis in waves [26] and maintain a specific distribution influenced by the density of epithelial cells [27]. The initial wave of DETCs originates from fetal thymic precursors [28] and populates the epidermis from birth [29]. Research has shown that DETCs have the ability to self-renew in the steady-state epidermis [30,31]. Despite this, our understanding of the fate of DETCs in both the steady-state and inflamed epidermes is limited [27,31].

Recent studies have explored the connection between the gut microbiome and γδ T cells in epithelial and non-epithelial tissues, such as the small intestine [32], lung [33,34], liver [35,36], gingiva [37], and testis [38,39]. While some research has looked into how the skin microbiota might affect skin γδ T cells [40,41], the direct relationship between DETCs and gut microbiota remains unclear.

In this study, we aimed to investigate the link between gut microbiota and DETCs. Specifically, we tested the hypothesis that microbial diversity influences the number of DETCs in the steady-state epidermis. The accumulated evidence indicates that gut microbiota can modulate systemic inflammation through immune-modulating metabolites, suggesting that changes in the gut microbiota might lead to systemic inflammation [42,43]. Given that γδ T cells in various tissues are regulated by gut microbiota, it is likely that DETCs are also influenced by gut microbiota or related systemic inflammation.

## 2. Material and Methods

### 2.1. Mice

C57BL/6 (B6) and Balb/c mice were obtained from Hyochang Science, Inc. (Daegu, Republic of Korea); specific pathogen-free (SPF)-housed Balb/c mice were kindly provided by Dr. Holzapfel (Handong Global University, Pohang, Republic of Korea). Unless otherwise stated, we used male mice at 8 weeks of age. The mice were housed either in non-barrier or barrier conditions at Handong Global University. All animal experiments were conducted following the guidelines and regulations of the Institutional Animal Care and Use Committee (IACUC) at Handong Global University, and the study protocols were approved under the reference numbers HGU-IACUC 20210518-005, HGU-IACUC 20220530-04, HGU-IACUC 20230719-11.

### 2.2. Induction of Inflammation in Mouse Ear Skin

Inflammation in the mouse ear skin was induced using two different protocols: (1) DNFB-induced inflammation: the mouse ears were topically treated with 20 μL of 0.5% 1-fluoro-2,4-dinitrobenzene (DNFB; Sigma-Aldrich, St. Louis, MO, USA) in a mixture of acetone and sunflower oil (1:4, *v*/*v*); (2) UV-C light-induced inflammation: the entire body of the mouse, including the ears, was exposed to UV-C light at a wavelength of 253.7 nm for a total duration of 30 min, resulting in a cumulative dose of 24.12 kJ.

### 2.3. Isolation of Epidermal Sheets

Epidermal sheets were isolated as described previously [44]. Mouse ears were dissected into dorsal and ventral halves, and the tissue was incubated in PBS containing 0.02 M EDTA (Sigma-Aldrich) for 60 min at 37 °C to facilitate the separation of the epidermis from the dermis.

### 2.4. Immunofluorescence Microscopy

Isolated epidermal sheets were fixed overnight in 100% ice-cold acetone. Following fixation, the sheets were washed once with phosphate-buffered saline (PBS) and then blocked with a 1% bovine serum albumin (BSA; Sigma-Aldrich) solution (200 μL) at room temperature (RT). For staining, the epidermal sheets were incubated for 30 min at 4 °C with specific antibodies: the fluorescein isothiocyanate (FITC)-labeled rat anti-I-A/I-E antibody (clone: M5/114.15.2) for the detection of Langerhans cells (LCs) and the AlexaFluor 488-labeled Armenian Hamster anti-γδ TCR antibody (clone: GL3) for the detection of DETCs. Additionally, all samples were counterstained for all hematopoietic cells using a phycoerythrin (PE)-labeled rat anti-mouse CD45 antibody (clone: 30-F11). The mounted samples were prepared using ProLong™ Gold Antifade Mountant with DAPI (Thermo Fisher Scientific, Waltham, MA, USA). The antibodies were obtained from BioLegend (San Diego, CA, USA). Immunofluorescence microscopy was performed using a Carl Zeiss Axio Imager a2 microscope (Oberkochen, Germany), and the acquired images were processed using ImageJ software v1.53k (National Institutes of Health and the Laboratory for Optical and Computational Instrumentation, Madison, WI, USA). We analyzed at least 10 fields from three mice in each experiment.

### 2.5. Flow Cytometry

The flow cytometry analysis of epidermal leukocytes was conducted following a previously described protocol [44]. Epidermal sheets were isolated and digested in PBS containing 0.1% collagenase I (Lakewood, NJ, USA) to obtain a single-cell suspension [44]. The cells were fixed with 4% paraformaldehyde in PBS (15 min, 4 °C) and permeabilized with 1% saponin (Sigma-Aldrich) in PBS (15 min, 4 °C). Subsequently, the cells were stained with the following antibodies (20 min, 4 °C): anti-AlexaFluor 488-labeled rat anti-human Langerin (CD207) (clone: 929F3.01; Dendritics, Lyon, France), PE-labeled Armenian hamster anti-mouse γδ TCR (clone: GL3), PerCP/Cy5.5-labeled rat anti-mouse CD45 (clone: 30-F11), PE/Cy7-labeled rat anti-mouse/human CD11b (clone: M1/70), and allophycocyanin (APC)-labeled anti-mouse CD3ε (clone: 145-2C11; Thermo Fisher Scientific). All antibodies were obtained from BioLegend (San Diego, CA, USA). The flow cytometry analysis was performed using Attune NXT (Thermo Fisher Scientific) or BD LSRii (BD Biosciences, Franklin Lakes, NJ, USA) flow cytometers. The data obtained were analyzed using the FlowJo software version 10.8 (BD Biosciences).

### 2.6. 16S rRNA Gene Amplicon Sequencing

The library preparation was performed according to the standard instructions of the 16S Metagenomic Sequencing Library Preparation protocol (IlluminaTM, Inc., San Diego, CA, USA). The V3–V4 regions of the bacterial 16S rRNA genes were amplified using aliquots of the isolated DNA from each sample. The V3–V4 region was amplified using the 341F-805R primers: 16s-341F: 5′-TCGTCGGCAGCGTCAGATGTGTATAAGAGACAGCCTACGGGNGGCWGCAG-3′; 16s-805R: 5′-GTCTCGTGGGCTCGGAGATGTGTATAAGAGACAGGACTACHVGGGTATCTAATCC-3′.

The sequences were obtained on the Illumina MiSeq platform in a 2 × 300 bp paired-end run.

### 2.7. Analysis of Sequencing Data

Reads were sorted using unique barcodes for each PCR product. The barcode, linker, and primer sequences were then removed from the original sequencing reads. Any reads containing two or more ambiguous nucleotides, those with a low-quality score (average score < 25), or reads shorter than 300 bp, were filtered out. Potential chimeric sequences were detected using the Bellerophon method [45].

### 2.8. Determination of Operational Taxonomic Units (OTU) and Taxonomic Classification

The pre-processed reads obtained from each sample were utilized for calculating the number of operational taxonomic units (OTUs). The clustering of the sequences from each sample was performed using a 97% sequence identity cut-off [46,47] using the QIIME software (version 1.8.0; Northern Arizona University, Flagstaff, AZ, USA). Taxonomic abundance was determined using the Ribosomal Database Project (RDP) Classifier (version 1.1; Michigan State University, East Lansing, MI, USA) with a confidence threshold of 0.8, utilizing the pre-processed reads from each sample. To normalize the microbial composition, the taxonomy abundance count was divided by the number of pre-processed reads for each respective sample. The Shannon index was employed to measure species richness (alpha diversity).

### 2.9. Metagenome Prediction and Metabolic Reconstruction of 16S rRNA Datasets

The metagenomes were predicted from the 16S rRNA data using the Phylogenetic Investigation of Communities by Reconstruction of Unobserved States (PICRUSt) tool [48]. To predict the metagenomes, closed reference OTU picking was performed against the Greengenes database (version 13.5; http://greengenes.lbl.gov, accessed on 30 May 2023). The resulting OTU table was then normalized using the normalize_by_copy_number.py script. The metagenomes were further predicted from the copy number-normalized 16S rRNA data using the predict_metagenomes.py script, specifically against the Kyoto Encyclopedia of Genes and Genomes (KEGG) database, utilizing the PICRUSt tool.

### 2.10. Statistical Analysis

The Mann–Whitney U test was employed for statistical analyses. *p*-values greater than 0.05 were deemed to indicate significant differences. Statistically significant *p*-values are denoted as follows: * *p* < 0.05, ** *p* < 0.01, and *** *p* < 0.001.

## 3. Results

### 3.1. Balb/c Mice Exhibit an Increase in DETC Numbers During Induced Skin Inflammation

**Figure 1:**
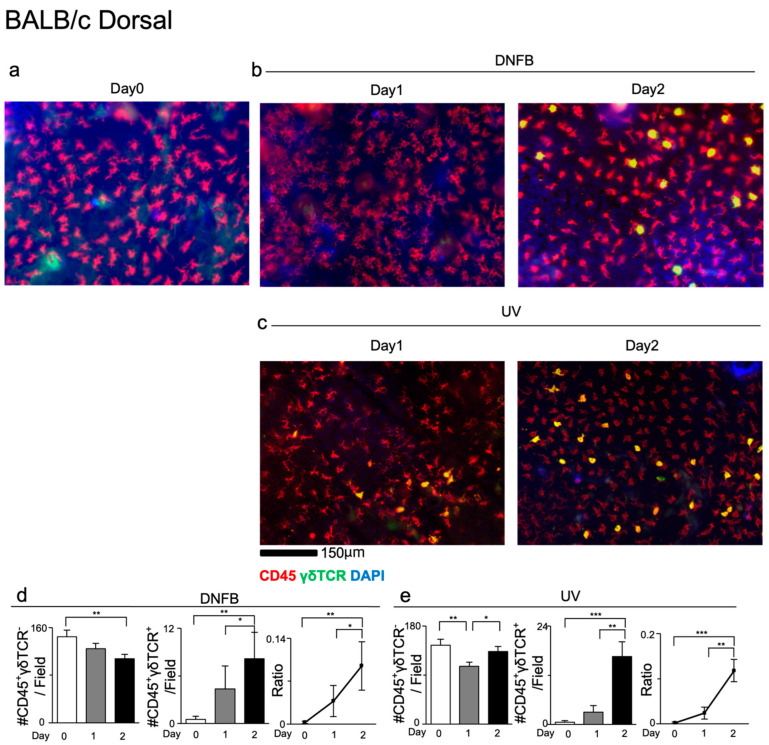
The numbers of DETCs are markedly increased after DNFB or UV treatment. (**a**–**c**) Immunofluorescence images of dorsal ear sheets of Balb/c mice (**b**) treated with DNFB or (**c**) irradiated with UV. CD45 (red), γδTCR (green) and DAPI (blue). (**d**,**e**) Bar graphs showing the numbers and of LCs and DETCs in the dorsal epidermal sheet counted at 0, 1 and 2 days after UV or DNFB treatment, as well as the ratio of DETCs to LCs. *n* = 13–14 areas from 3 mice. * *p* < 0.05, ** *p* < 0.01, *** *p* < 0.001. The data represent mean ± SEM. Statistics analyzed by Mann–Whitney U test.

First, we sought to understand how DETCs respond to locally induced skin inflammation. Therefore, we examined the impact of local skin inflammation on DETCs in Balb/c mice using two methods. Of note, Balb/c mice have lower baseline numbers of DETCs in the ear epidermis compared to B6 mice, resembling humans in this feature [49] (Figure 1a and Figure S1).

To study the impact of local skin inflammation on DETC dynamics, we sensitized the mouse ear with hapten DNFB or exposed the mice to UV light for 30 min. We collected the ear skin epidermis on days 1 and 2 after sensitization or UV exposure and stained the samples for CD45 and γδTCR. CD45^+^γδTCR^+^ cells represented γδ T cells while CD45^+^γδTCR^-^ cells represented Langerhans cells (LCs). A microscopic examination revealed that LC numbers decreased slightly in the case of topical DNFB application but remained relatively stable upon UV exposure. In contrast, DETC numbers increased continuously over the two days. As a result, the ratio of DETCs to LCs gradually increased (Figure 1d,e). Collectively, our data indicated that induced skin inflammation significantly enhances the abundance of DETCs in Balb/c mice.

### 3.2. DETC Number Varies Based on Housing Conditions

**Figure 2:**
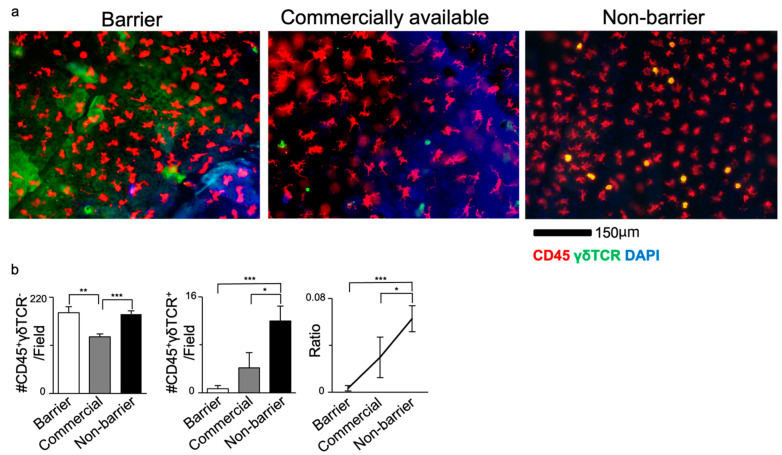
Non-barrier-housed Balb/c mice display higher numbers of DETCs compared to barrier-housed mice. (**a**) Immunofluorescence staining of CD45 (red), γδTCR (green) and DAPI (blue). (**b**) Bar graphs showing the number of LCs and DETCs in the dorsal epidermal sheet, as well as the ratio of DETCs to LCs. The data represent mean ± SEM. *n* = 10–12 areas from 3 mice. * *p* < 0.05, ** *p* < 0.01, *** *p* < 0.001. The data represent mean ± SEM. Statistics analyzed by Mann–Whitney U test.

We observed that local inflammation increases the numbers of DETCs in the skin. Next, we examined whether different housing conditions affected DETC numbers. We compared the ear skin of Balb/c mice maintained under SPF conditions (‘barrier-housed’) with those housed in the non-barrier facility (‘non-barrier-housed’). The epidermal sheets were stained for γδTCR and CD45, with CD45^+^γδTCR^+^ cells identified as DETCs (Figure 2a). Additionally, we included commercially obtained Balb/c mice in our analysis and compared them to our barrier- and non-barrier colonies. Notably, the ear epidermis of commercially obtained mice had a higher number of DETCs compared to our barrier-housed mice, but fewer compared to the non-barrier-housed mice. In contrast, only minor differences in the number of LCs were observed among the three groups (Figure 2b). Taken together, our data suggested that environmental factors and housing conditions significantly impact DETC populations in the skin.

### 3.3. Supplementation with Bedding from a Non-Barrier Environment Increased DETC Numbers in SPF Mice

Our data demonstrated the occurrence of DETCs increases in a non-barrier environment. To investigate whether the differences in gut microbial environment contribute to this variation, we transferred Balb/c mice from SPF to a quarantine facility and maintained them there for several months. We divided Balb/c mice into two groups: one group received clean bedding weekly, while the other group was given a mixture of non-barrier dirty bedding and clean bedding at a 1:1 ratio initially, which was then renewed by 50% every week thereafter.

**Figure 3:**
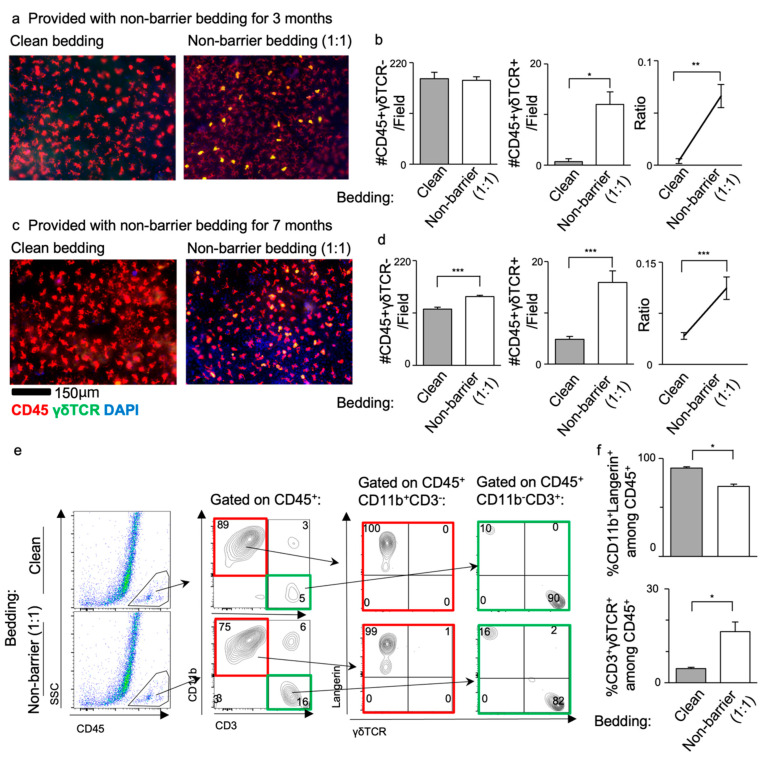
Balb/c mice displayed increased DETC numbers when exposed to non-barrier bedding. Quantitative analysis of LCs and DETCs in the dorsal epidermis of Balb/c after receiving either cleaning or partial non-barrier bedding for (**a**,**b**) three or (**c**,**d**) seven months. (**a**,**c**) Immunofluorescence staining of CD45 (red), γδTCR (green) and DAPI (blue). (**e**) Representative dot plots showing the gating strategy used to define LC and DETC populations. Contour plots showing the frequency of CD11b^+^, CD3^+^, γδTCR^+^ cells in the epidermal sheet with the frequency of CD11b^+^Langerin^+^ LCs and CD3^+^γδTCR^+^ DETCs among CD45^+^ cells. (**f**) Quantitative analysis of LC and DETC frequencies using flow cytometry. The data represent mean ± SEM. *n* = 12 areas from 3 mice. * *p* < 0.05, ** *p* < 0.01, *** *p* < 0.001. The data represent mean ± SEM. Statistics analyzed by Mann–Whitney U. SSC—side scatter.

We analyzed the two groups at three and seven months after the transfer from SPF to quarantine. At three months, the immunostaining of ear sheets revealed that Balb/c mice exposed to the mixed dirty non-barrier bedding had a higher percentage of DETCs among epidermal leukocytes, compared to those maintained with clean bedding only (Figure 3a). The proportion of LCs remained unchanged (Figure 3b).

To assess the impact of aging on DETC numbers, we examined whether the duration of exposure to a dirty atmosphere affected DETC activation. After an additional four months in quarantine, we compared the mean numbers of LCs and DETCs, and the DETC-to-LC ratio. Interestingly, the mean number of LCs did not differ significantly between mice exposed to dirty non-barrier bedding and those maintained with clean bedding only, while the mean number of DETCs and the DETC-to-LC ratio were significantly higher in mice exposed to a dirty non-barrier bedding. A similar trend in DETC dynamics was observed in both groups quarantined for seven months (Figure 3b,d). This suggests that the duration of exposure is not key to DETC occurrence.

A flow cytometry analysis further revealed an approximately 3.5-fold increase in the frequency of CD3^+^γδTCR^+^ DETCs in mice upon exposure to the dirty non-barrier bedding, while CD11b^+^Langerin^+^ LCs decreased (Figure 3e,f).

Taken together, our combined data indicate that DETC occurrence is influenced by the gut microbiota and environmental conditions, rather than the duration of exposure alone. This highlights the relationship between the gut microbial environment and DETC dynamics in the skin.

### 3.4. Gut Microbiota Analysis Revealed Distinct Microbial Composition and Functional Profiles Between Different Housing Conditions

**Figure 4:**
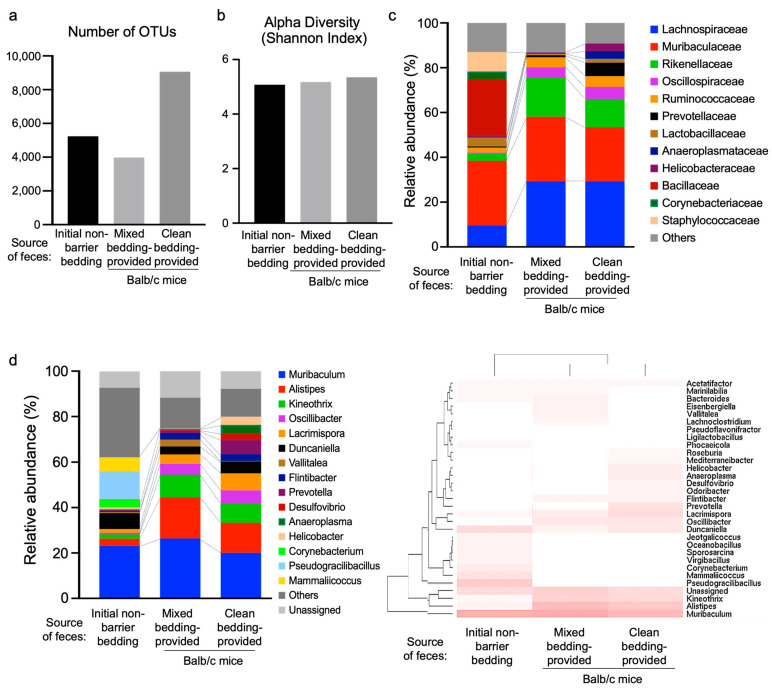
Metagenomic comparison of different housing conditions. (**a**) Analysis of operational taxonomic unit (OTU) number. (**b**) Comparison of the Shannon index of different groups. (**c**) Taxonomic distribution of bacterial compositions at the family level. (**d**) Taxonomic distribution and hierarchical clustering of bacterial compositions at the genus level.

The activation of DETCs in response to environmental conditions, as observed in the epidermal cell composition analysis, prompted us to investigate the potential influence of housing conditions on gut microbiota. To compare the gut microbiota composition under different housing conditions, we conducted 16S rRNA metagenome analyses with fecal samples preserved from the initial non-barrier bedding and those collected from mice provided with mixed non-barrier plus clean bedding (‘mixed bedding’) or clean bedding only (‘clean bedding’) (Figure 4).

The sequencing of the hypervariable V3 + V4 regions of 16S rRNA genes allowed us to obtain operational taxonomic units (OTUs) at a minimum similarity threshold of 97% (Figure 4a). Additionally, the alpha diversity was determined using the Shannon index (Figure 4b). Of note, we observed changes in the murine gut microbiome at the family and genus levels (Figure 4c,d). At the family level, *Muribaculaceae* (Gram-negative) and *Lachnospiraceae* (mostly Gram-positive) were predominantly present in all groups, while the relative proportions of these taxa shift across bedding types. *Bacillaceae* was uniquely observed in the initial non-barrier bedding (Figure 4c). At the genus level, *Muribaculum* emerged as the most abundant species across all groups (Figure 4d). Overall, non-barrier bedding seems to have a higher relative abundance of Gram-negative bacteria compared to clean bedding, which shows more Gram-positive taxa. Mixed bedding appears to have an intermediate composition, with both Gram-negative and Gram-positive bacteria present but not as distinctly skewed as the other conditions. Species more abundant in the clean group or the initial non-barrier group were identified. (Table 1). Conversely, species such as *Muribaculum*, *Bacteroides*, *Phocaeicola*, and *Vallitalea* were more abundant in the mixed bedding and initial non-barrier groups (Table 2). Notably, the initial non-barrier group exhibited a distinct pattern compared to the clean and mixed bedding groups, with some genera exclusively present in the initial non-barrier group (Table 3). Notably, we observed the presence of *Bacillaceae* in the feces from the non-barrier group, which correlated with increased numbers of DETCs, whereas *Prevotellaceae* were found exclusively in the feces from the clean group, associated with lower numbers of DETCs. Interestingly, *Bacillaceae* have been previously described to enhance the population of intestinal γδ T cells, while *Prevotellaceae* were found to reduce their numbers [50].

**Figure 5:**
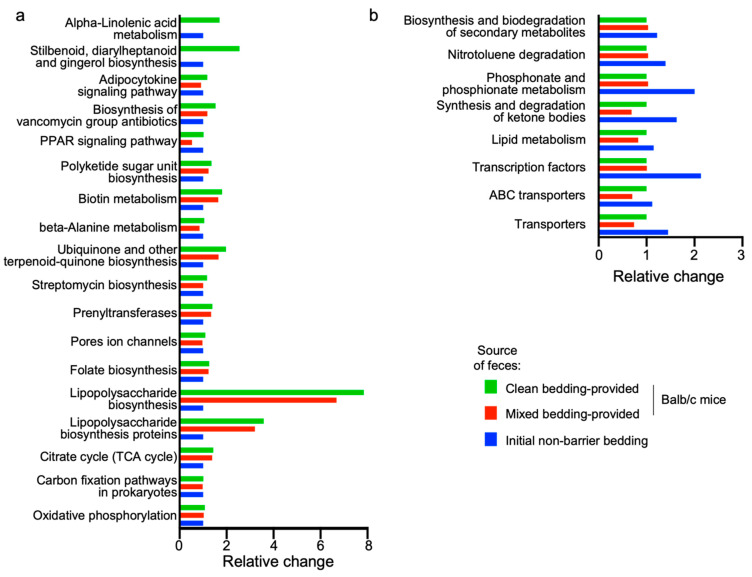
Functional composition of the microbial community metagenome as determined by PICRUSt analysis. (**a**) Functional pathways prominent in the fecal samples from mice provided with clean bedding. (**b**) Functional pathways prominent in the fecal samples collected from initial non-barrier ‘dirty’ bedding.

To characterize the functional profiles of the colonic microbiota, we employed PICRUSt analysis in conjunction with the Kyoto Encyclopedia of Genes and Genomes (KEGG) database (Figure 5a,b). This analysis compared functional differences among all housing conditions. We focused on pathways that were at least 150% more abundant in the feces from mice provided with clean bedding only, as compared to those from the initial non-barrier bedding (Figure 5a), and vice versa (Figure 5b). Intriguingly, the pathways prominent in feces from the clean bedding group were also upregulated in mice with mixed bedding, compared those from the initial non-barrier bedding. These pathways include ‘biosynthesis of vancomycin group antibiotics’, ‘polyketide sugar unit biosynthesis’, ‘biotin metabolism’, ‘ubiquinone and other terpenoid-quinone biosynthesis’, ‘streptomycin biosynthesis’, ‘prenyltransferases’, ‘folate biosynthesis’, ‘lipopolysaccharide biosynthesis’, ‘lipopolysaccharide biosynthesis proteins’, ‘citric acid cycle’, and ‘oxidative phosphorylation’ (Figure 5a). In contrast, pathways related to ‘biosynthesis and biodegradation of secondary metabolites’, ‘nitrotoluene degradation’, ‘phosphonate and phosphonate metabolism’, and ‘transcription factors’ were most prominent in the initial non-barrier bedding group, followed by the mixed bedding group, and was least prominent in the clean bedding group (Figure 5b).

Overall, these results revealed the distinct microbial composition and relative abundance of bacterial families and genera among the different housing conditions. Additionally, a PICRUSt analysis provided insights into the functional profiles of the colonic microbiota, with specific pathways enriched in the clean bedding and initial non-barrier bedding groups. These findings collectively demonstrate a link between housing conditions, DETC activation, and the gut microbiota, highlighting the relationship between the skin and gut immune responses in the context of environmental factors.

## 4. Discussion

This study aimed to elucidate the relationship between gut microbiota and DETCs. Our findings reveal that local skin inflammation leads to a significant increase in DETC numbers in Balb/c mice. We also demonstrate that DETCs can be activated by environmental factors, as shown by our epidermal cell composition analysis. Investigating the gut microbiota under varying housing conditions further unveiled distinct microbial compositions and functional profiles, highlighting a potential link between housing conditions, DETC activation, and the gut microbiota. This implies the complex interplay between skin and gut immune responses in response to environmental stimuli. One limitation of this study is the exclusion of skin microbiome analysis. The mice were treated by transferring feces-containing bedding and given that skin bacteria can migrate freely within the facility, coupled with the absence of overt skin phenotypes, we did not prioritize skin microbiome analysis. While the skin microbiome is generally less diverse than the gut microbiome, composed of a few dominant species adapted to local conditions, we cannot entirely rule out its potential involvement in the observed immune responses.

Our interest in DETCs originated from the observation of their complete absence in Balb/c mice raised in germ-free (GF) conditions. However, the low number of DETCs in Balb/c mice currently limits our ability to further explore the molecular mechanisms involved. In future studies, we plan to adopt a single-cell transcriptome analysis with enriched DETC populations to address these questions and gain a deeper understanding of the interactions between gut microbiota, DETCs, and skin immune responses.

Recent studies have suggested intriguing insights for the abundance of γδ T cells over αβ T cells in the epidermis and dermis of young mice kept in SPF conditions. For instance, *herpes simplex* virus (HSV)-infected mice developed HSV-specific CD8^+^ T cells with a dendritic morphology that displaced local DETCs [6]. Similarly, *C. albicans*-infected mice exhibited a large population of CD4^+^ tissue-resident memory (TRM) T cells that outnumbered dermal γδ cells and dominated IL-17 production upon re-challenge [51]. In environments rich with pathogens, such as pet stores or barns, mice experience repeated antigenic challenges, leading to a diminished density of skin γδ T cells in mice housed in pet stores or living in barns, and αβ T cells are far more abundant, as are memory T cells. The immune systems of these mice more closely resemble those of adult humans, whereas those of SPF mice resemble the immune systems of neonatal humans. One could speculate that skin γδ T cells represent a primitive adaptive immune system that is replaced by αβ TRM cells over time as the mouse encounters more and more antigenic pathogens through the skin.

Several studies have demonstrated a reduction in γδ T cells in GF mice, compared to their SPF counterparts. Specifically, Vγ6^+^ uterine γδ T cells are decreased in GF mice, relative to their SPF controls [52]. Similarly, Vγ4^+^ peripheral IL-17-expressing γδ T cells show variable reductions in GF mice, compared to SPF mice [37]. Additionally, decreased DETC levels have been observed in SPF mice or in those experiencing dysbiosis [53]. These findings suggest that the presence of certain bacterial strains is critical for the maintenance of γδ T cells.

Our study identified *Bacillaceae* in the feces of the non-barrier group, which exhibited higher DETC counts, and *Prevotellaceae* in the clean group, which had lower DETC counts. In line with this, gut-residing *Bacillaceae* have previously been reported to increase intestinal γδ T cell numbers, while *Prevotellaceae* have been associated with their decrease [50]. This indicates that these bacterial strains may exert systemic effects on γδ T cells beyond their local intestinal environment, also impacting DETCs. Further investigations in GF models are needed to validate these findings.

## 5. Conclusions

The gut microbiome, comprising trillions of microorganisms residing in our gastrointestinal tract, plays a pivotal role in both physiology and pathology. Over the years, it has been shown to communicate with various organs, including the small intestine, lung, liver, gingiva, and testis, thereby modulating their functions. However, the relationship between the gut microbiome and the skin epidermis has remained unexplored.

In this study, we investigated whether local skin inflammation and systemic microbial alterations influence the population of DETCs, the predominant immune cell type in the skin epidermis. Our findings revealed that the abundance of DETCs is shaped not only by local skin inflammation but also by changes in gut microbiome composition. Notably, an increased number of DETCs correlated with a rise in *Bacillaceae* and a decline in *Prevotellaceae* populations.

These observations indicate the need for further validation using gnotobiotic models in GF settings. Additionally, future research should investigate the role of the skin microbiome in regulating DETCs.

## Figures and Tables

**Table 1:** Abundant genus in the clean bedding group.

Bacterial Genus	Relative Abundance (%)		
	Non-Barrier	Mixed	Clean
*Lacrimispora*	1.38	3.53	6.42
*Prevotella*	0.43	0.85	5.30
*Oscillibacter*	0.60	4.11	4.86
*Anaeroplasma*	0.05	0.34	3.11
*Helicobacter*	0.72	0.20	3.07
*Flintibacter*	0.47	2.47	2.58
*Desulfovibrio*	0.11	0.39	2.57
*Odoribacter*	0.16	0.11	2.17
*Mediterraneibacter*	0.19	0.27	1.63
*Roseburia*	0.75	0.38	1.55

**Table 2:** Abundant genus in the non-barrier and mixed bedding group.

Bacterial Genus	Relative Abundance (%)		
	Non-Barrier	Mixed	Clean
*Muribaculum*	20.40	22.65	17.11
*Bacteroides*	1.99	2.47	0.91
*Phocaeicola*	1.25	0.56	0.43
*Vallitalea*	0.44	2.59	0.17

**Table 3:** Relative abundance of non-barrier-only genus.

Bacterial Genus	Relative Abundance (%)
*Pseudogracilibacillus*	10.94
*Mammaliicoccus*	5.39
*Corynebacterium*	3.05
*Jeotgalicoccus*	2.52
*Oceanobacillus*	2.50
*Sporosarcina*	2.33
*Virgibacillus*	2.11
*Enteractinococcus*	1.78
*Ornithinibacillus*	1.62
*Cerasibacillus*	1.48
*Lentibacillus*	1.38
*Atopostipes*	1.19

## Data Availability

The raw data were generated at Handong Global University and are available from the corresponding author, Jea-Hyun Baek, upon request.

## Supplementary Materials

The following supporting information can be downloaded at: https://www.mdpi.com/article/10.3390/life14121695/s1, Figure S1. The epidermal ear skin epidermis of C57Bl6 harbors only two immune cell components, namely CD3^+^γδTCR^+^ γδ T cells and Langerin+CD11b+ Langerhans cells. (a,b) Immune cell composition in the ear skin epidermis of C57Bl/6 shown by (a) immunostaining and (b) flow cytometry.

## Abbreviations

CD; cluster of differentiation; DETC, dendritic epidermal T cell(s); DNFB, 1-fluor-2,4-dinitrobenzene; γδTCR, gamma-delta T cell receptor; GF, germ-free; LC, Langerhans cell(s); PICRUSt, Phylogenetic Investigation of Communities by Reconstruction of Unobserved States; rRNA, ribosomal RNA, SPF, specific pathogen-free; TCR, T cell receptor; TNF, tumor-necrosis factor; UV, ultra-violet
