# Supplementary material for: Gut Microbiota Regulates the Homeostasis of Dendritic Epidermal T Cells
Source: Life (Basel). 2024 Dec 21;14(12):1695. doi: 10.3390/life14121695 (PMC11677426; doi:10.3390/life14121695)
Supplement: Supplementary file 1 [file life-14-01695-s001.zip › life-3272536-supplementary.pdf]

B6 Dorsal

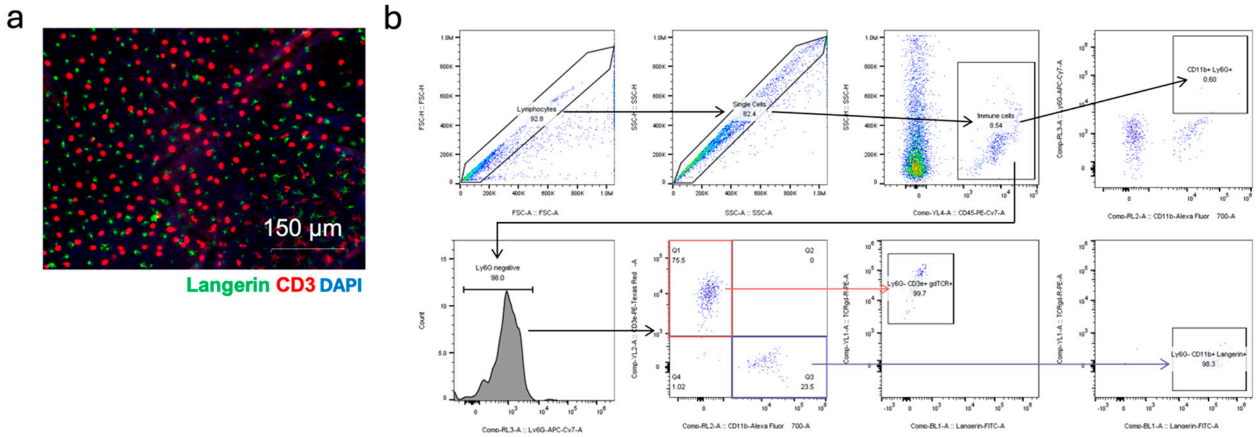

Supplemental Figure S1. The epidermal ear skin epidermis of C57Bl6 harbors only two immune cell components, namely CD3^{+}γδTCR^{+} γδ T cells and Langerin+CD11b+ Langerhans cells. (a ,b) Immune cell composition in the ear skin epidermis of C57Bl/6 shown by (a) immunostaining and (b) flow cytometry.
